# Deep learning–based vortex decomposition and switching based on fiber vector eigenmodes

**DOI:** 10.1515/nanoph-2023-0202

**Published:** 2023-06-15

**Authors:** Mengdie Hou, Mengjun Xu, Jiangtao Xu, Jiafeng Lu, Yi An, Liangjin Huang, Xianglong Zeng, Fufei Pang, Jun Li, Lilin Yi

**Affiliations:** The Key Lab of Specialty Fiber Optics and Optical Access Network, Joint International Research Laboratory of Specialty Fiber Optics and Advanced Communication, Shanghai University, Shanghai 200444, China; College of Advanced Interdisciplinary Studies, National University of Defense Technology, Changsha 410073, China; Nanhu Laser Laboratory, College of Advanced Interdisciplinary Studies, National University of Defense Technology, Changsha 410073, China; State Key Laboratory of Advanced Optical Communication Systems and Networks, Shanghai Jiao Tong University, Shanghai 200240, China

**Keywords:** deep learning, mode decomposition, vector eigenmodes, vortex switching

## Abstract

Structured optical fields, such as cylindrical vector (CV) and orbital angular momentum (OAM) modes, have attracted considerable attention due to their polarization singularities and helical phase wavefront structure. However, one of the most critical challenges is still the intelligent generation or precise control of these modes. Here, we demonstrate the first simulation and experimental realization of decomposing the CV and OAM modes by reconstructing the multi-view images of projected intensity distribution. Assisted by the deep learning–based stochastic parallel gradient descent (SPGD) algorithm, the modal coefficients and optical field distributions can be retrieved in 1.32 s within an average error of 0.416 % showing high efficiency and accuracy. Especially, the interference pattern and quarter-wave plate are exploited to confirm the phase and distinguish elliptical or circular polarization direction, respectively. The generated donut modes are experimentally decomposed in the CV and OAM modes, where purity of CV modes reaches 99.5 %. Finally, fast switching vortex modes is achieved by electrically driving the polarization controller to deliver diverse CV modes. Our findings may provide a convenient way to characterize and deepen the understanding of CV or OAM modes in view of modal proportions, which is expected of latent applied value on information coding and quantum computation.

## 1 Introduction

Cylindrical vector (CV) and optical vortex beams with orbital angular momentum (OAM) have received widespread attention from many researchers due to their unique polarization and phase singularity [1–3]. Driven by the potential applications of CV beams in laser machining [4], super-resolution microscopy [5, 6], data storage [7] and sensing [8, 9], etc., active methods of generating a single CV or OAM field with high purity have become preferred in the experiment. One popular method among these techniques is based on the free-space optical components, including Q-plates [10, 11], spatial light modulators (SLMs) [12], and spiral phase plates [13], which always require high-precise optic alignment to generate a single CV or OAM beam. As an example, the input light polarization relative to its fast axis using the Q-plates is strictly chosen for azimuthally or radially polarized beams. By comparison, another is to deliver CV or OAM modes based on the vector eigenmodes in the fibers by using all-fiber mode converters, such as long period gratings (including helical gratings) [14–23] and mode selective couplers (MSCs) [24–29], a single high-order linearly polarized (LP) mode can be converted from the fundamental mode (LP_01_), but easily influenced by the amplitudes and phase difference due to slightly different propagation constants of degenerated CV modes in the step-index fibers. Actually, the electrical fields of high-order modes (HOMs) consist of linear superposition of CV modes, such as the LP_*m*1_ mode (*m* ≥ 1), which have been understood well and exploited to decompose four-fold degenerated vector eigenmodes with different polarization distributions.

The LP or circularly polarized (CP) OAM modes in the fibers can also be formulated by means of coherent superposition of these vector eigenmode bases. As far as we know, there is still a challenge to obtain a single CV or OAM mode with a high purity both in the free space or fiber waveguide. Early, Shapira et al. [30] presented a noninterfering approach to achieve complete mode decomposition (MD) for light fields in the optical waveguide. In 2020, Mao et al. [31] reported an interference method to calculate the proportions of degenerated modes, which considered all possible modes in multimode fibers. Recently, Borhani et al. [32] recovered the phase of the input image by training the deep neural networks. Wang et al. [33] achieved the intelligent recognition of different OAM mode bases in the ring-core fiber by the deep learning technique, and the recognition rate is close to 93.3 %. It is noted that deep learning techniques, particularly convolutional neural network (CNN) algorithm, have performed MD of LP modes in optical fibers and stand out for global search ability and excellent real-time performance [34–37]. The MD of the LP modes are achieved by reconstructing the mode intensity, and the average correlation is 0.9912 [38]. The CNN-based optimized algorithm is also used to design ultra-compact silicon photonic devices with less training data [39]. In addition, some techniques for single-shot measuring vector modes have been proposed based on metasurfaces, such as single spin–decoupled metasurface and polarization-sensitive dielectric metalens. The vortex can be recognized by using a single azimuthal-quadratic phase metasurface-based photonic momentum transformation [40]. Imaging polarimetry is widely used for precise measurement of any polarized light using a crosstalk-free broadband achromatic full Stokes method [41]. An angular superoscillatory metalens is utilized for determining the OAM spectrum based on precalibrated mapping relationship [42]. On the other side, the stochastic parallel gradient descent (SPGD) algorithm with fast convergence ability and low measurement requirements has been extensively utilized for the MD of diverse spatially scalar LP modes in practical experiments [43], where the correlation function reaches 0.995 with time of 0.7 s [44]. Previously, the MD of the LP modes was researched widely by reconstructing single intensity distribution. However, precisely decomposing degenerated CV and OAM modes in the fiber has rarely been investigated in view of their polarization and phase distributions, simultaneously. Therefore, two crucial issues are raised, that is, the accurate measuring proportions and intelligent controlling of spatially varying CV or OAM modes, which play a pivotal role in improving the efficiency of mode control and optimizing the polarization properties of generated donut beams. Since CV and OAM beams are characterized by spatially varying polarization and phase distribution, it is of great importance to propose a vector-eigenmode decomposition method for recovering the optical field and retrieving the proportions of each group of CV and OAM modes.

Generally, CV and OAM modes as two degenerated mode bases in the fibers hold a transformation relation. Previous researches have revealed that a single CP-OAM mode is composed of two CV modes and a single LP-OAM mode consists of four CV modes. Moreover, the complete transformation relation of arbitrary high-order modes has also been derived, which can even provide a new perspective for the expression of the most generally elliptical polarized OAM modes [45, 46]. In this case, an unidentified mode can also be expressed in OAM mode bases when the corresponding modal coefficients of CV modes are obtained by the MD of the degenerated CV modes, which has not been reported. Intuitively, a new hybrid first-order Poincaré sphere (PS) [47] may be constructed and introduce an extra dimension to describe spatial modes with arbitrary proportions in that two groups of the CV or OAM modes are located on two first-order PSs, respectively. So far, traditional fiber-squeezing devices are used to change the proportions and polarizations of the spatial modes in fibers, which is difficult to generate desired and switchable polarization states due to manual error. Here, the electrically driven polarization controller (EPC) is utilized to achieve a qualitative control of the CV or OAM beams, which would be immensely useful for intelligent polarization search of donut beams.

In this paper, we successfully and precisely recovered the complex amplitudes of degenerated modes (CV, LP-OAM, and CP-OAM) by reconstructing a multi-view image due to the polarization degeneracy of the degenerated spatial modes. Based on the deep learning–based SPGD algorithm, the decomposed results can be obtained within 1.32 s and the average error of modal coefficients is nearly 0.416 %. It is worth noting that evitable phase ambiguity caused by the SPGD algorithm may result in different modal proportions in OAM mode bases. However, the interference patterns are exploited to verify the phase distribution, which can also be determined by checking the direction of elliptical polarization. Furthermore, it is of paramount importance to distinguish the circular polarization direction when a single CP-OAM mode is considered. The experimentally generated donut beams are decomposed in the CV, LP-OAM, and CP-OAM mode bases, indicating that CV modes with purity of 99.5 % are realized. Employing the input voltages of the EPC, the CV modes are selected at a speed of 4 s. Therefore, fast vortex mode switching can be achieved from different inputs of CV modes passing through wave plates with fixed angles. It is believed that the proposed approach of decomposing and switching CV and OAM modes may facilitate applications of singular CV or OAM beam in fields, such as laser beam characterization, dipole orientation of single-photon sources for quantum optical communication, and fiber-optic communication exploiting multiple spatial modes.

## 2 Principle and simulation results

### 2.1 Principle of MD for degenerated fiber vector eigenmodes

Most optical fibers for practical applications are guiding light in the weakly guiding limit of Δ = (*n*_1_ − *n*_2_)/*n*_2_ << 1, Δ is the relative index difference between the core (*n*_1_) and the cladding (*n*_2_). The number of guided modes in the fiber is dependent on the physical dimensions of the core/cladding regions, refractive index, and light wavelength. The guiding electric fields in fibers can be described as the eigen-solutions of the Helmholtz equation in the cylindrical waveguides, which are the intrinsic vector modes that can propagate stably in fibers. The CV solution in the step-index fiber can be presented by the Bessel function *J*_*m*_(*hr*) of the first or second kinds. Considering each CV mode in a cylindrical fiber is characterized by two mode indices *m* and *n*, where the azimuthal mode index m refers to the angular dependence and *n* is the radial mode index. Under the weakly guiding approximation, many CV modes are nearly degenerate, and LP fields can be formed by linear combinations of these nearly degenerate CV modes whose propagation constants are almost the same. For example, the LP_01_ mode consists of ${HE}_{11}^{\text{even}}$ and ${HE}_{11}^{\text{odd}}$ mode, and the first high-order LP_*mn*_ is composed of TM_01_, TE_01_, ${HE}_{21}^{\text{even}}$ , ${HE}_{21}^{\text{odd}}$ modes (*m*, *n* = 1), and of ${HE}_{m+1,1}^{\text{even}/odd}$ , ${EH}_{m-1,1}^{\text{even}/odd}$ for higher-order LP_*mn*_ (*m* > 1, *n* = 1). All LP modes can be constructed from linear combinations of different basic fiber modes.

Generally, the spatial mode field from the few-mode fiber (FMF) can be decomposed as the four degenerated CV modes with arbitrary complex amplitudes assuming a single LP_*mn*_ mode dominated in the fiber. To retrieve the modal coefficients of the four degenerated CV eigenmodes, the original and reconstructed near-field light fields should be compared from both spatially variant polarization and intensity distributions. Thus, the modal field distributions of a single LP_*mn*_ mode in the fiber can be expressed as (*m* ≥ 1, *n* = 1):

(1) $$E_{\text{in}}=\overset{4}{\underset{k=1}{\sum }}\rho_{k}e^{\text{i}\theta_{k}}E_{k}$$

where *ρ*_*k*_ and *θ*_*k*_ are the corresponding modal amplitude and phase of four CV eigenmodes ( ${HE}_{m+1,1}^{\text{even}/odd}$ , ${EH}_{m-1,1}^{\text{even}/odd}$ ).

*E*_*k*_ is their normalized electric fields. Here, *θ*_1_ is set to 0 as a phase reference. Thus, the corresponding modal weight $\rho_{k}^{2}$ can be obtained with the given normalized condition of $\sum_{1}^{4}\rho_{k}^{2}=1$ and relative phase *θ*_*k*_ (*k* = 2,3,4) can be calculated. Furthermore, the theoretical model of the first-order PSs intuitively reveals all polarization states and mode profiles to generate arbitrary CV beams. Each point on the PSs can be described by the Stokes parameters through a QWP and a polarizer, which is expressed by the following equations [47]:

(2) $$\left\{\begin{array}{c} S_{1}=\left(I_{0{}^{\circ}}-I_{90{}^{\circ}}\right)/S_{0}\;\\ S_{2}=\left(I_{45{}^{\circ}}-I_{-45{}^{\circ}}\right)/S_{0}\;\\ S_{3}=\left(I_{\text{Rcp}}-I_{\text{Lcp}}\right)/S_{0}\; \end{array}\right.$$

where *I*_0°_, *I*_45°_, *I*_90°_, and *I*_−45°_ stand for the light intensities through a polarizer at the corresponding angles. *I*_Rcp_ and *I*_Lcp_ denote the intensities of light by using a QWP at 45° and −45°, respectively, and *S*_0_ is the intensity distribution out of the FMF. TM_01_, TE_01_, ${HE}_{21}^{\text{even}}$ , and ${HE}_{21}^{\text{odd}}$ modes are located on the equator of the ±1st-order PSs, which suggests that the polarization state of any superimposed CV modes can be characterized by *S*_1_ and *S*_2_ with *S*_3_ = 0. Therefore, the mode field with the group base of CV eigenmodes in arbitrary proportion can be derived for the four projected polarization intensities through a linear polarizer, which at the specific polarization *ψ* can be expressed as [31]

(3) $$E_{\text{pol}}=M_{\text{pol}}E_{\text{in}}=\left[\begin{array}{cc} cos^{2}\psi & \sin\;\psi \;\cos\;\psi \\ \sin\;\psi \;\cos\;\psi & \;sin^{2}\psi \end{array}\right]E_{\text{in}}.$$

**Figure 1:**
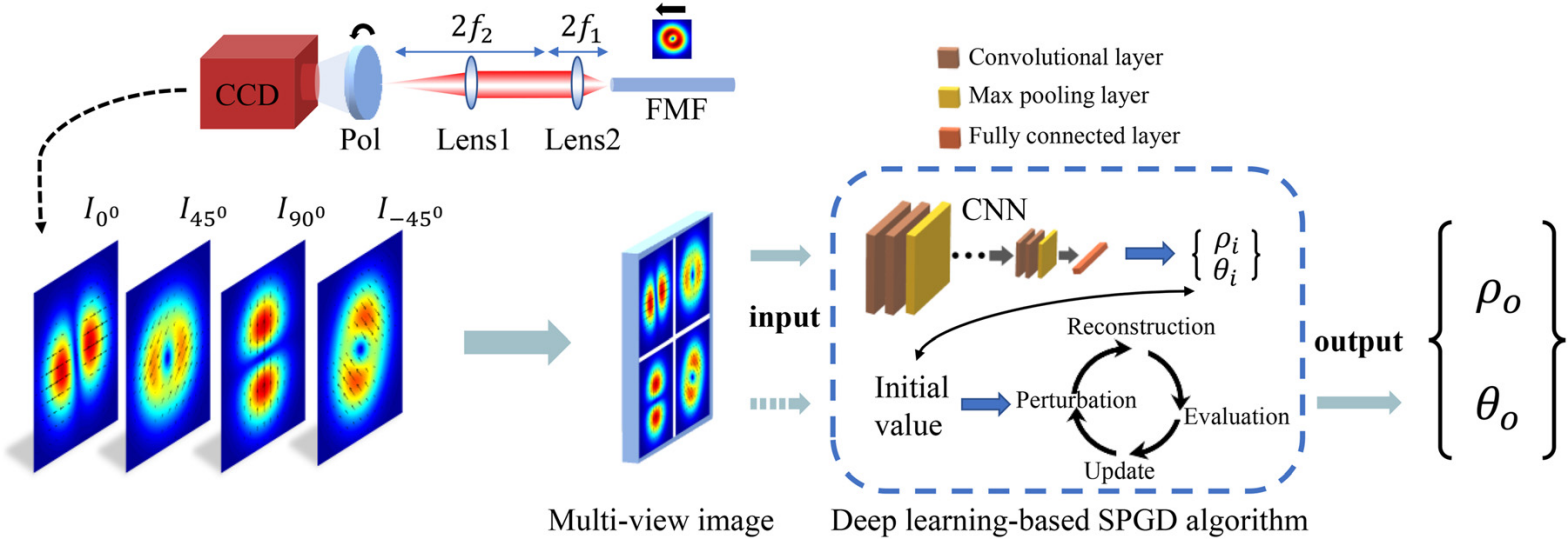
Overview of deep learning–based SPGD algorithm for decomposing four eigenmodes. CCD, charge-coupled device; Pol., linear polarizer; Lens1, Lens2, focal lens; FMF, few-mode fiber.

According to the theoretical derivation of Eq. (2), it can be found that the modal fields of any CV mode can be obtained by calculating the intensity distributions at *ψ* = 0°, 45°, 90°, and −45°. The MD schematic of CV modes is shown in Figure 1. The output modal patterns from the fiber are imaged to a charge-coupled device (CCD) camera through a 4f system and projected at the polarization angles of four by rotating the polarizer. Specifically, four grayscale images, as multiple perspectives, are reshaped into a multi-view image for training the CNN model. It is noted that the multi-view image covers the polarization characteristics of the CV modes. The deep learning–based SPGD algorithm is composed of a well-trained CNN model and the SPGD algorithm. During the test procedure, the randomly generated multi-view image is fed into the CNN, and then the rough global values of modal coefficients (*ρ*_*i*_ and *θ*_*i*_) expressed in CV mode bases can be predicted with a small margin of error. The SPGD algorithm can be divided into five parts: initializing the variables, generating perturbations, reconstructing the patterns, evaluating the merit function, and updating the variables. Here, the initial variables (*ρ*_*i*_ and *θ*_*i*_) for the SPGD algorithm are replaced by the output vector from the CNN to shorten the time of exploring exact modal weights (*ρ*_*o*_) and relative phases (*θ*_*o*_).

### 2.2 Deep learning–based SPGD algorithm

In order to decompose four CV modes accurately, the pretrained VGG-16 model is firstly adapted by using the multi-view images. The CNN model is mainly divided into three layers, such as convolution layers, the max pooling layers, and the fully connected layers. More details of the CNN model were described in ref. [38]. In the training process, 40,000 beam patterns expressed in CV mode bases with the resolution of 128 × 128 are generated with random label coefficients. Through calculating polarization projected images by use of a rotating polarizer, corresponding 40,000 multi-view images data with the resolution of 256 × 256 are reshaped, as shown in Figure 1. Around 80 % of the beam patterns is exploited as the training data for training the model, 10 % of which is used for validating the performance of the model during the training period. The remaining 10 % is employed as the testing data to generate the initial modal coefficients for the subsequent SPGD algorithm. Since the input training image is a one-channel image data, the filter size of the first convolution layer is changed from 3 × 3 × 3 to 3 × 3 × 1. The dimension of the output vector of the last fully connected layer is modified to 2*N* − 1, where *N* is the number of modes (here, *N* = 4 for CV modes). In a word, the output vector size is equal to the label size. Modal weights and relative phases of CV modes are used for the label vector. Besides, the softmax and classification layers are replaced by the sigmoid and regression layers for achieving the regression, respectively.

**Figure 2:**
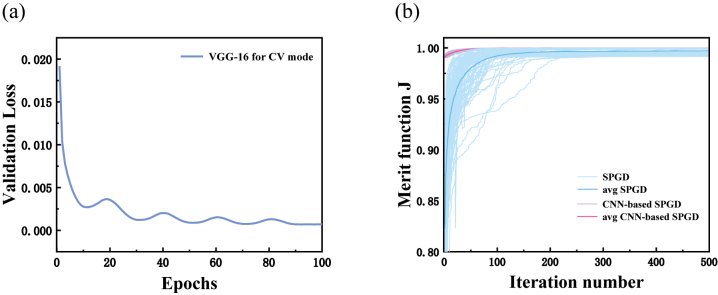
Performance for decomposing CV modes based on VGG-16. (a) The validation loss for the CV mode using VGG-16. (b) The merit function curve (fine) and the average curve (thick) based on 100 times by using two algorithms.

The validation loss function is utilized to estimate the degree of consistency between the predicted and real modal coefficients, which is expressed by the mean-square error (MSE) as:

(4) $$Loss=\overset{M}{\underset{i=1}{\sum }}\overset{2N-1}{\underset{k=1}{\sum }}\frac{{\left(y_{i,\text{o}}\left(k\right)-y_{i,\text{p}}\left(k\right)\right)}^{2}}{M}$$

where *y*_*i*,o_ and *y*_*i*,p_ represent the original and predicted label vector, which consists of the modal weights and phases. *M* is the batch-size and is set as 10 for training all the training data. Here, Adam optimizer with the initial learning rate of 5 × 10^−5^ is exploited. In addition, the learning rate is renewed by using the cosine annealing learning rate every 20 epochs where the minimum learning rate is set to 10^−5^. The validation loss curve in Figure 2(a) indicates that training process of can converge after 88 epochs, which suggests that the feature of the multi-view images can be learned well with low loss and fast converge speed.

The essence of the deep learning–based SPGD algorithm is to minimize the polarization projected difference between the reconstructed electric field (*I*_re_) and the presupposed one (*I*_pre_), which has higher computational precision and can be utilized for solving the optimization constraints. The iteration process of the deep learning–based SPGD algorithm is briefly described as follows: (1) the rough globally optimized values (*ρ*_*i*_ and *θ*_*i*_) of the presupposed multi-view image can be obtained by the well-trained model, which are further set as the initial values of the SPGD algorithm. (2) Generating random perturbations (*δρ*_*i*_ and *δθ*_*i*_) to change the variables to acquire temporary reconstructed fields (*I*_re±_). (3) Evaluating the merit functions of correlation between the measured and presupposed intensities, which can be described as

(5) $$J=\left|\frac{\int \;\int \left(I_{\text{re}}-\bar{I_{\text{re}}}\right)\left(I_{\text{pre}}-\bar{I_{\text{pre}}}\right)rdrd\varphi }{\sqrt{\int \;\int {\left(I_{\text{re}}-\bar{I_{\text{re}}}\right)}^{2}rdrd\varphi \int \;\int {\left(I_{\text{pre}}-\bar{I_{\text{pre}}}\right)}^{2}rdrd\varphi }}\right|.$$

(4) Updating the variables in step (2) for the next iteration based on the difference of the merit function *J*.

It is worth noting that compared to the residual intensity, *J* has lower noise sensitivity, which can be adopted as an important evaluation criterion for MD. When *I*_pre_ = *I*_re_ is satisfied, the predicted (*ρ*_*o*_ and *θ*_*o*_) and the original modal coefficients are equal in the ideal case. To exhibit the excellent performance of the deep learning–based SPGD algorithm, firstly, the average merit function for decomposing the CV modes by using these two algorithms based on 100 times are described in Figure 2(b). It can be found that the average merit function *J* of the MD by using these two algorithms are 0.997 and 0.999, respectively. It can be found that the efficiency for obtaining the accurate results is higher by the deep learning–based SPGD algorithm. Secondly, the average errors of the modal weight (∆*ρ* = | $\rho_{\text{pre}}^{2}-\rho_{\text{re}}^{2}$ |) and relative phase (∆*θ* = ||*θ*_pre_| − |*θ*_re_||/2*π*) by using these two algorithms can be obtained. The average errors reach 0.608 % and 0.416 % based on 100 times iteration using the CNN-based SPGD algorithm within 1.32 s, respectively. It is worth noting that the error of modal coefficients can reach 0.004 % in one time iteration. However, two mean errors by the SPGD algorithm are 6.585 % and 10.69 %. In a word, the CNN can indeed enhance the ability to explore the optimization solutions. It can be concluded that our proposed scheme not only can shorten the calculation time but also can obtain more precise decomposed results, which paves the way toward the real-time and batch processing.

### 2.3 Decomposing CV and OAM modes enabled by deep learning

**Figure 3:**
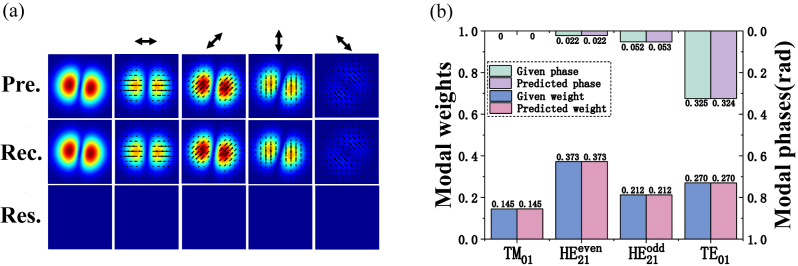
One typical decomposition result of CV modes by the deep learning-based SPGD algorithm. (a) The presupposed and reconstructed beam intensities of a hybrid CV mode, and residual intensities are shown for comparison by the deep learning–based algorithm. The angle of the polarizer is marked by the bidirectional arrow. (b) The corresponding given and predicted modal coefficients. Pre.: presupposed patterns. Rec.: reconstructed patterns. Res.: residual patterns.

Here, we have demonstrated that the modal weights and phases in the CV modes with any proportions can be confirmed by the deep learning–based SPGD algorithm. In order not to lose the generality, one typical MD case of a mixed CV mode is presented in Figure 3. The simulated total intensity distribution and four polarization projection patterns of the presupposed and reconstructed beams are illustrated in Figure 3(a). Moreover, the residual intensity distributions also indicate the presupposed beam patterns and the reconstructed ones are in great similarity. Furthermore, the beam pattern at 45° polarization is almost equal to the total light field, indicating that 45° polarization occupies a large proportion, which can be constructed by combing the ${LP}_{11}^{\text{even}}$ and ${LP}_{11}^{\text{odd}}$ mode with the same proportions. Moreover, the ${LP}_{11}^{\text{even}}$ and ${LP}_{11}^{\text{odd}}$ modes can be represented by TM_01_ + ${HE}_{21}^{\text{even}}$ and ${HE}_{21}^{\text{odd}}$ + TE_01_, respectively. The corresponding modal coefficients are shown in Figure 3(b); it can be seen that predicted modal weights and phases are almost equal to the given ones, which are consistent with the field distributions and the theoretical analysis.

It should be noted that the CV modes with the same modal weights but opposite phases have equal polarized intensity distributions because there exists the inevitable phase ambiguity. The noteworthy thing is that the predicted modal phases here are the absolute values relative to the phase of the first mode (TM_01_). That is to say, there exist two possible mixed CV modes located on the southern and northern hemispheres of the hybrid first-order PS with same S_1_, S_2_ but opposite S_3_.

The helical phase of OAM modes is an important feature, which also plays an essential role in the modal weight and phase of CV modes. The interference pattern is typically needed to further confirm the phase information. Therefore, the modal coefficients expressed in OAM mode bases can also be predicted well with the additional interference patterns. Many studies have found the transformation relation among CV modes, LP-OAM ( $\bar{x}OAM_{\pm 1}$ , $\bar{y}OAM_{\pm 1}$ ) and CP-OAM (*σ* ^±^OAM_±1_) modes, which has been described in ref. [45, 46]. *σ* ^−^ and *σ* ^+^ represent the left-handed and right-handed circular polarization, and $\bar{x}$ and $\bar{y}$ are horizontal and vertical linear polarization, respectively. It can be found that two coefficient matrices are nonsingular. Here, column vectors are arbitrary complex vectors expressed in OAM and CV mode bases. In a word, there is only one group of LP-OAM or CP-OAM modes corresponding with any weighted CV modes, and vice versa. For the elliptical polarization, there could exist two set of modal phases in the CV mode bases, which is opposite of each other with the same modal weights. In this case, two groups of modal coefficients expressed in OAM mode bases could also be obtained. Thus, by observing the interference fringe, the exact phases can be determined further, which is beneficial to ensure the modal weights of OAM modes and the exact phase in the CV and OAM mode bases.

**Figure 4:**
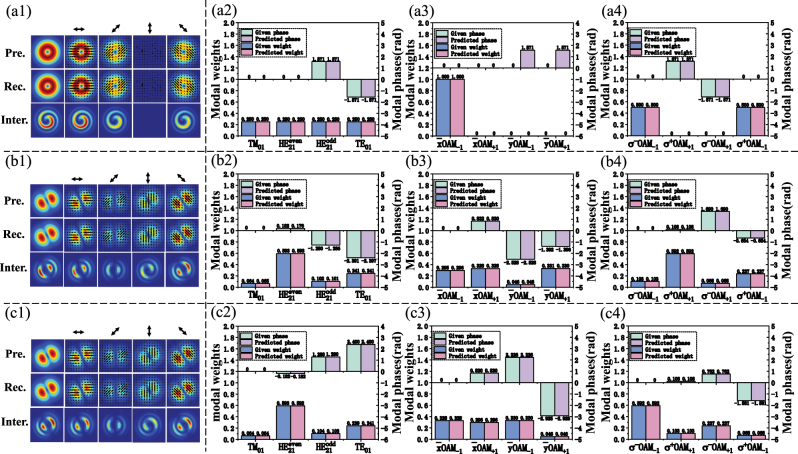
Three typical reconstructed intensity distributions and predicted modal coefficients by the deep learning–based SPGD algorithm, and the presupposed intensities and given modal coefficients are represented for comparison. (a1)–(a4): a single $\bar{x}OAM_{-1}$ mode; (b1)–(b4) and (c1)–(c4) are two hybrid OAM modes with same intensity distributions but different interference patterns. Pre.: presupposed patterns. Rec.: reconstructed patterns. Inter.: interference patterns. The angle of the polarizer is marked by the bidirectional arrow.

Firstly, the decomposition results of a single LP-OAM mode are introduced as shown in Figure 4(a1)–(a4). Note that the reconstructed beam patterns match well with the presupposed ones almost at every polarization, which indicates the high accuracy of the MD method and also implies the feature of a single $\bar{x}OAM$ . Moreover, the OAM beams are also interfered with a referenced spherical Gaussian beam in order to confirm the feature of helical phase structure. Comparing the interference patterns, $\bar{x}OAM_{-1}$ mode with a clockwise vortex shape is selected as shown in Figure 4(a1), which is the same as the presupposed ones. Hence, a single $\bar{x}OAM_{-1}$ mode can be determined from its weight prediction as shown in Figure 4(a3), which agrees well with the presupposed beam patterns. Figure 4(a2) shows that the modal weights for predicting four CV modes are the same. Furthermore, there exists a ±*π*/2 time delay of ${HE}_{21}^{\text{odd}}$ and TE_01_, respectively. At the meantime, we can obtain *σ* ^−^OAM_−1_ and *σ* ^+^OAM_−1_ with the same proportion in Figure 4(a4). It represents that the $\bar{x}OAM_{-1}$ can be obtained through equal superposition of left-handed (*σ* ^−^) and right-handed (*σ* ^+^) CP-OAM modes with the same to topological charge (TC) of −1. In the same way, $\bar{y}OAM_{-1}$ can also be obtained by superposing two *σ* ^±^OAM_−1_ modes.

Secondly, the phase ambiguity of two mixed OAM modes can also be identified through the interference as shown in Figure 4(b1)–(b4) and (c1)–(c4). The total mode intensity and its four polarized distributions are the same but five interference patterns are slightly different. Taking 90° polarized intensities as an example, Figure 4(b1) and (c1) clearly show the counter-clockwise and clockwise vortex-liked patterns, respectively. From Figure 4(b2) and (c2), the modal weights expressed in CV mode bases are the same while the phases are opposite. Nevertheless, in the LP-OAM mode bases, there exist two different proportions in weights and phases as shown in Figure 4(b3) and (c3). To be specific, the modal weights are just exchanged, and the phases of the last two modes remain different, which can be derived from the transformation matrices. When the CV modes with opposite phases are predicted, ±*i* in the transformation matrix is understood to swap the weights of $\bar{x}OAM_{-1}$ , $\bar{x}OAM_{+1}$ and $\bar{y}OAM_{-1}$ , $\bar{y}OAM_{+1}$ , which further leads to different phases in the OAM mode bases. The combination of LP-OAM modes in Figure 3(b3) indicates that the OAM mode with TC of +1 occupies a larger proportion, which is consistent with the interference patterns. However, the superposition result in Figure 4(c3) shows the proportion in OAM modes with TC of −1 is larger, which can be reflected in Figure 4(c1). By the same token, we can get the same conclusions in CP-OAM modes as shown in Figure 4(b4) and (c4). These results demonstrate that opposite phases of CV modes can affect the proportions of OAM modes, although the polarized intensity patterns are the same. Therefore, the interference patterns should be beneficial and are of great importance to confirm the modal weights and phase of the OAM modes. Except the interference, the direction of elliptical polarization can also be utilized to determine the phase expressed in OAM mode bases by using the QWP.

Especially, for a single CP-OAM mode, it can be found that there is no intensity discrepancy among the four polarized donut beams. Another ambiguity of left-handed and right-handed polarization needs to be separated before using four polarized intensity patterns of the MD method. As discussed above, a single LP-OAM mode can be decomposed well by referencing the interference patterns. Since the QWP can provide a phase shift, the CP-OAM modes can be transformed into LP-OAM modes. Passing through a QWP whose optical axis coincides with the *y*-axis, *σ* ^−^OAM_−1_ mode is transformed into a −45° LP-OAM_−1_ mode while *σ* ^+^OAM_+1_ is converted to a 45° LP-OAM_+1_ mode. As for the −45° LP-OAM_−1_ mode, the brightest beam pattern appears at −45° (*I*_Lcp_), the darkest one at 45° (*I*_Rcp_), and medium ones at the other two angles of the polarizer. That is to say, *S*_3_ is equal to −1, and *S*_1_, *S*_2_ = 0. Therefore, the *σ* ^−^OAM_−1_ mode on the south pole of the −1st-order PS can be selected. Afterward, we can acquire the modal coefficients expressed in CV and LP-OAM modes. It can be found that $\bar{x}OAM_{-1}+i\bar{y}OAM_{-1}=\sigma^{-}OAM_{-1}$ . On the contrary, $i\bar{x}OAM_{+1}+\bar{y}OAM_{+1}=\sigma^{+}OAM_{+1}$ can be obtained. Therefore, a single CP-OAM mode can also be decomposed by transforming it into an LP-OAM mode with a QWP.

To conclude, we can acquire the exact modal weights of CV modes on the equator of the PS based on four polarized intensities, and the corresponding modal coefficients expressed in LP-OAM or CP-OAM mode bases can also be obtained through the matrices. As for the mixed CV modes on the hemispheres of the PS, the proportions in the three mode bases can be confirmed with additional detecting of the interference or direction of elliptical polarization. The two detections are both needed when one CP-OAM mode exists. In reality, the MD of OAM modes can be directly achieved by replacing the *E*_*k*_ in Eq. (1) with the Jones vectors of the OAM modes. More importantly, employing the matrices, the polarization relation among CV, LP-OAM and CP-OAM modes can be deduced and understood more easily from the perspective of the mode proportion.

## 3 Experimental results

### 3.1 Pattern recognition of CV and OAM modes

**Figure 5:**
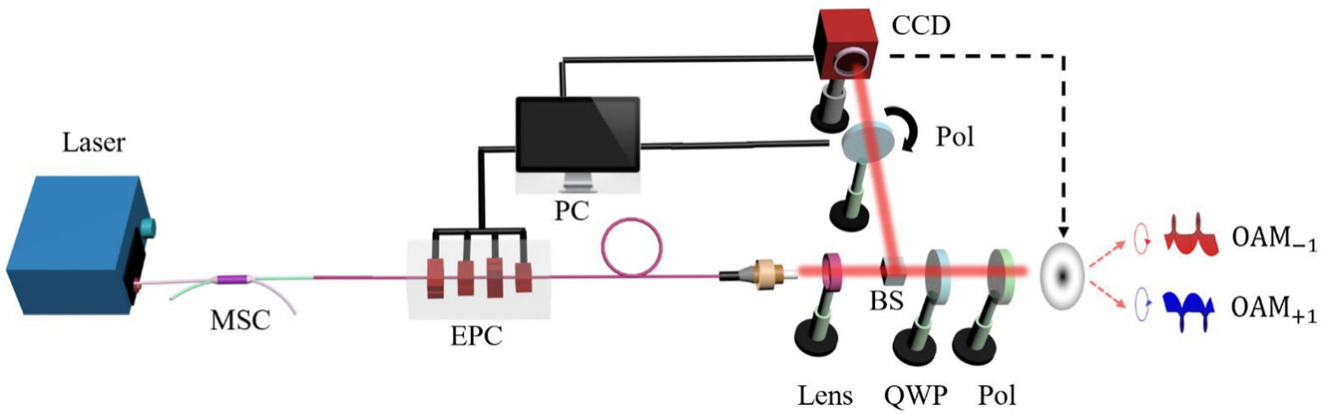
Experimental setup to decompose four degenerated modes and achieve dynamic mode switching between vortex and CV modes. MSC, mode selective coupler; EPC, electrically driven polarization controller; Lens, focal lens system; BS, nonpolarization beam splitter; Pol, linear polarizer; CCD, charge-coupled device; QWP, quarter-wave plate; PC, personal computer.

**Figure 6:**
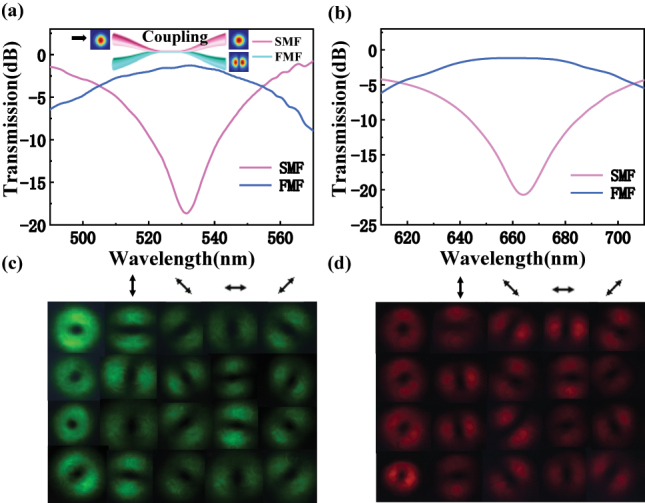
The schematic and characterization of MSCs. (a) The measured transmission spectra of the MSC at 532 nm; the inset represents the schematic of the MSC composed of an SMF and FMF. (b) The measured transmission spectra of the MSC at 660 nm. Polarization characteristic of CV beams (TM_01_, TE_01_, ${HE}_{21}^{\text{even}}$ , and ${HE}_{21}^{\text{odd}}$ ) excited by MSCs at (c) 532 nm and (d) 660 nm.

The schematic of the experimental setup is illustrated in Figure 5, which can be separated into three parts: mode conversion module, MD module, and field control module. The mode conversion module is utilized to transform the LP_01_ mode into specific *l*th order modes. As an all-fiber mode converter, two home-made MSCs working at visible wavelengths are generally used to achieve LP_11_ mode with high purity and broad bandwidth. The MSC made of a single-mode fiber (SMF) and an FMF is shown as the inset in Figure 6(a). To match their propagating constants, the diameters of two fibers in the fused region need to be appropriately chosen. The core diameter of the step-index FMF is 19.2 μm for propagating the HOMs. The detailed coupling principle and fiber parameters of the MSCs have been introduced in ref. [28]. Briefly, different order LP modes with any coupled ratio by the MSCs have been obtained by a two-step process, i.e., pretapering the SMF to adjust its diameters and fusing together with the FMF to optimize the output high-order LP modes.

The measured transmission spectra of the MSCs delivering LP_11_ mode working at the central wavelength of 532 nm and 660 nm are shown in Figure 6(a) and (b), respectively. The dips of the spectra indicate that the mode conversion efficiency is over 15 dB (∼97 %) and the measured 3 dB bandwidth is over 10 nm. By carefully adjusting the pressure and torsion angle of the PC clamped to the FMF, the degeneracy of LP_11_ mode is eliminated so that the desired vector modes can be excited. The polarization characteristics of CV beams at the wavelength of 532 nm and 660 nm are shown in Figure 6(c) and (d). The first column is the near-field of CV beams without the polarizer, and the later four columns are the intensity distributions along with the rotating direction (0°, −45°, 90°, and 45°, marked by the bidirectional arrow) of the linear polarizer.

As depicted in Figure 5, a laser source at the wavelength of 660 nm is launched into an MSC with the output power ratio of 90:10, where two arms are used to be a high-order mode and reference beam for vortex interference. Here, higher-order modes than LP_11_ mode in the FMF can be ignored due to the high purity of the MSC, which indicates that the first-order CV or OAM mode is mainly considered for the MD experimentally. The 90 % port of the FMF is connected to an in-line all-fiber EPC (EPC-400, OZ Optics), which is utilized to redistribute four degenerated vector eigenmodes of LP_11_ mode propagating in the fiber by changing their relative amplitudes and phases by means of stress-induced birefringence [48]. The EPC employing the mechanically squeezing technique is controlled through four-channel input DC voltages by varying over a ±5 V range, which enables over 25 *N* force applied to the fiber to introduce sufficient birefringence and phase changing the polarization over 360° in scale. Therefore, any desired output polarization on the first-order PSs can be achieved through the EPC. A focal lens (denoting a 4-f imaging system mentioned above) is placed closely after the EPC output and utilized to collimate the output beams. After passing through a nonpolarization beam splitter (BS), the beam is divided into two branches for the purpose of the MD and vortex switching process. A rotatable linear polarizer is set to 0°, 45°, 90°, and −45°, respectively, and the intensity patterns at four corresponding polarization angles are recorded by a CCD (400–1100 nm, Ophir-Spiricon). Four projected polarization patterns are arrayed to be a multi-view image before sending to the deep learning–based SPGD algorithm for the MD procedure. The searching time for the accurate global optimization values can be 1.32 s as previously mentioned, exhibiting much better real-time performance than the SPGD algorithm.

**Figure 7:**
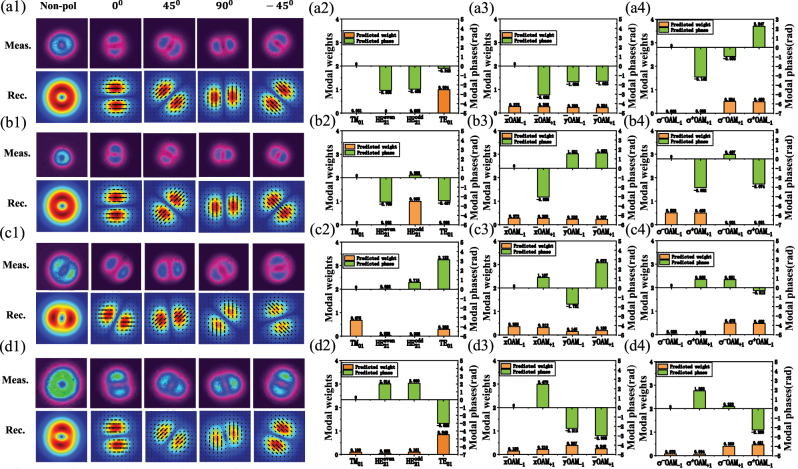
The experimentally measured and reconstructed intensity patterns of (a1) TE_01_, (b1) ${HE}_{21}^{\text{odd}}$ , (c1) TM_01_–TE_01_, and (d1) a hybrid CV mode by using the deep learning–based algorithm, and the angles of the polarizer are relative to *x*-axis. The predicted modal coefficients of the measured modes expressed (a2, b2, c2, d2) in CV mode bases; (a3, b3, c3, d3) in LP-OAM mode bases; (a4, b4, c4, d4) in CP-OAM mode bases.

The measured intensity patterns and decomposed results of TE_01_, ${HE}_{21}^{\text{odd}}$ , TM_01_–TE_01_, and a hybrid vector mode by the proposed algorithm are illustrated in Figure 7(a)–(d), respectively. It is found that four measured polarized beam profiles have great consistency with the reconstructed ones. Additionally, the predicted modal weights of TE_01_ and ${HE}_{21}^{\text{odd}}$ modes are over 99 % as described in Figure 7(a1, a2) and (b1, b2), indicating the generation of a single CV beam with high purity. Moreover, according to the transformation relations between CV and OAM modes, a single CV mode separated as four LP-OAMs or two CP-OAMs is presented and decomposed with high accuracy, as predicted in Figure 7(a3, a4) and (b3, b4). It can be seen that TE_01_ and ${HE}_{21}^{\text{odd}}$ modes are composed of four LP-OAM modes with almost the same amplitudes but exactly opposite phases in Figure 7(a3) and (b3). It should be highlighted that those CV states located on the ±1st-order PSs can be seen as the superposition of two orthogonal *σ* ^+^OAM_−1_, *σ* ^−^OAM_+1_ and *σ* ^−^OAM_−1_, *σ* ^+^OAM_+1_, respectively. Besides, TE_01_ mode is located on +1st-order PS, while ${HE}_{21}^{\text{odd}}$ mode on −1st-order PS. Consequently, these two CV modes can be expressed in the CP-OAM modes as shown in Figure 7(a4) and (b4). The predicted weights and phases are consistent with those from the theory.

As for the hybrid TM_01_ and TE_01_ modes as shown in Figure 7(c) and (d), the predicted modal weights of TM_01_ and TE_01_ modes are 0.675 and 0.292 with a phase difference of *π*. The corresponding expressions under LP-OAM and CP-OAM mode bases are described in Figure 7(c3) and (c4). Another hybrid CV mode can be expressed in CV and OAM mode bases as shown in Figure 7(d2)–(d4), where TE_01_ mode accounts for a large proportion (∼84.6 %). From the beam patterns in Figure 7(d1), it can also be found that the polarization of the hybrid mode is close to TE_01_ mode, but their weights and phase are clearly distinguished. It is worth noting that some measured CV modes are on the equator of the PS, which suggests that phase ambiguity in corresponding CV modes is not considered.

It is noted from the results of Figure 7 that projected polarization intensities are accurately predicted with measured ones by using the multi-view images in the algorithm, but the reconstructed mode patterns may present slightly inconsistent due to the following reasons: (1) the projected mode intensity using CCD acquisition is limited by its intensity pixels and measured resolution; (2) the presence of other LP modes in the fibers may also add the intensity perturbation; and (3) the CNN model for predicting CV mode is trained by the simulated data rather than experimental data. The prediction accuracy can be greatly improved considering these reasons in the future work. In general, the predicted coefficients expressed in CV and OAM mode bases are self-consistent with the measured beam patterns. Consequently, the modal decomposition of CV and OAM modes based on our scheme remains a high prediction performance in the actual experiments.

### 3.2 Fast vortex mode switching

The field control module shown in Figure 5 is used to generate specific CV beams and achieve different vortex switching. Currently, driving an EPC by programming the applying voltages is to change the mode output from the MSC. Since the EPC has four channels to change the voltages, we set the time interval between different voltage groups as 4 s. Actually, the interval can be shorter because the EPC can respond at a rapid response speed within microseconds. When the optimized voltage values are found, the dynamic and automatic switching among different CV beams can be realized.

**Figure 8:**
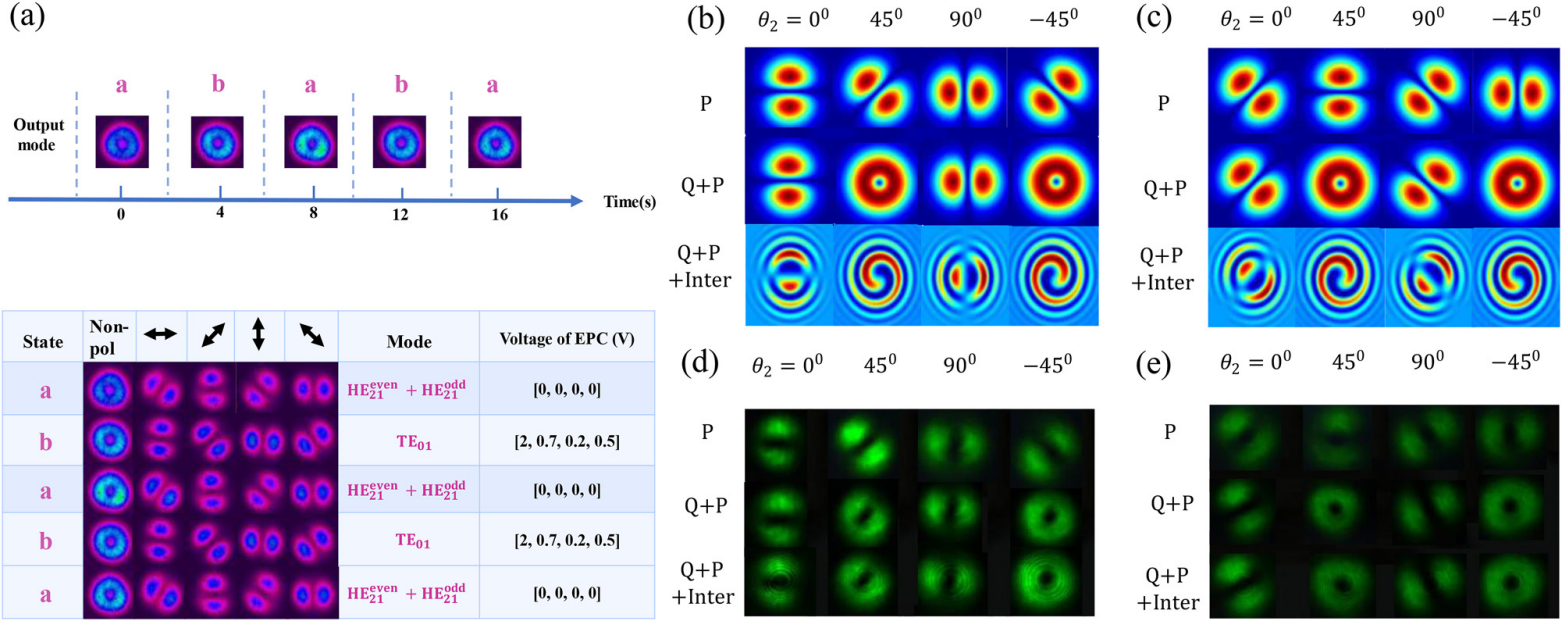
Vortex mode switching based on CV modes. (a) The dynamic transverse mode switching (above) and polarization distributions (below) of ${HE}_{21}^{\text{even}}+{HE}_{21}^{\text{odd}}$ , TE_01_ modes modulated by the EPC. The angle of the polarizer is marked by the bidirectional arrow. (b) Simulated intensity and interference patterns after a QWP and a polarizer (*θ*_2_) for TE_01_, ${HE}_{21}^{\text{even}}$ + ${HE}_{21}^{\text{odd}}$ modes. (d) And (e) are experimental results corresponding to (b) and (c), respectively.

As presented in Figure 8(a), two donut-shaped modes labeled by the state **a** ( ${HE}_{21}^{\text{even}}+{HE}_{21}^{\text{odd}}$ ) and **b** (TE_01_) modes, respectively, can be switched every 4 s by driving the EPC. The intensity profiles and polarization states under the invariant voltages are almost identical, indicating the high robustness and memorability of the EPC. In addition, the switching interval can be shorter, which demonstrates that fast and accurate CV mode switching certainly provides great potential in programmable optical sources.

Meanwhile, it is known that CV modes with arbitrary order *l* can be considered as a linear combination of two CP-OAMs with opposite TC (*l* ≥ 1). Moreover, four CV modes consist of two CP-OAMs with opposite TCs. The QWP can provide a phase shift and turn the circular polarization into linear polarization as pointed out above. Thus, a single CV mode can be transformed into LP-OAM_±1_ in two projection positions through the QWP with a specific angle. Then the polarizer can select two LP-OAM modes at two orthogonal angles, respectively. The fast axis angle relative to the *x*-axis of the QWP is *θ*_1_ and the transmission angle to the *x*-axis of the polarizer is *θ*_2_. The Jones matrices of the QWP and the polarizer can be represented by $H_{\text{QWP}}\left(\theta_{1}\right)$ and $H_{\text{pol}}\left(\theta_{2}\right)$ , respectively. The detailed equations have been described in ref. [49, 50]. The Jones matrix of the combined QWP and the polarizer can be written as *H*_S_(*θ*_1_, *θ*_2_). Here, we take *l* = 1 as an example. When four special CV modes are used as the inputs, we can theoretically calculate four output OAM modes, which are expressed as

(6) $$\begin{array}{ll} H_{\text{S}}\left(90{}^{\circ},\pm 45{}^{\circ}\right)\times \left\{\begin{array}{c} \begin{array}{c} \text{T}{\text{M}}_{01}\\ \text{T}{\text{E}}_{01}\\ {\text{HE}}_{21}^{\text{even}} \end{array}\\ {\text{HE}}_{21}^{\text{odd}} \end{array}\right\} & =\frac{e^{\frac{i\pi }{4}}F_{11}\left(r\right)k}{2}\left[\begin{array}{c} \;1\\ \pm 1 \end{array}\right]\;\times \left\{\begin{array}{c} \begin{array}{c} e^{\mp \text{i}\varphi }\\ e^{\mp \text{i}\varphi }\\ e^{\pm \text{i}\varphi } \end{array}\\ e^{\pm \text{i}\varphi } \end{array}\right\} \end{array}$$

where $F_{11}\left(r\right)$ represents the radial field distribution, and *k* is a constant. It is worth noting that the CV modes here are generalized, that is, some mixed CV beams including TM_01_ ± TE_01_ and ${HE}_{21}^{\text{even}}\pm {HE}_{21}^{\text{odd}}$ modes can get the similar results.

Based on the analysis above, the vortex mode switching at the wavelength of 532 nm using a QWP and a polarizer is achieved. The experimental results agree well with the simulations as illustrated in Figure 8(b)–(e). The previous generated CV beams by adjusting the EPC, TE_01_, and ${HE}_{21}^{\text{even}}+{HE}_{21}^{\text{odd}}$ are used as the inputs to achieve the vortex switching. When the QWP is parallel to the optical axis of the polarizer, the transmitted patterns are consistent with CV beams through one polarizer. When the angular difference is changed to ±45°, the donut beams appear and their interference profiles exhibit right-handed and left-handed helixes, which suggests that two OAMs with opposite TCs have been selected. It is noted that the TE_01_ mode is composed of *σ* ^+^OAM_−1_ and *σ* ^−^OAM_+1_ mode while ${HE}_{21}^{\text{even}}+{HE}_{21}^{\text{odd}}$ mode consists of *σ* ^+^OAM_+1_ and *σ* ^−^OAM_−1_. However, the polarization states of generated LP-OAMs with opposite TCs are oriented as the direction of ±45° LP. In addition, TE_01_ mode can be converted into OAM_∓1_ and ${HE}_{21}^{\text{even}}+{HE}_{21}^{\text{odd}}$ mode can be transformed into OAM_±1_ when *θ*_2_ is ±45°. Considering the previous simulation MD results of LP-OAM modes, it is known that the purity of the produced LP-OAMs can also be calculated by further recording four polarized beam patterns and the interference patterns. Therefore, fast and accurate mode control of CV and LP-OAM modes by using the programmable EPC provide a reliable and switchable donut light source, which can be applied in stimulated emission depletion microscopy (STED) [51] and super-resolution lithography [52].

## 4 Conclusions

In summary, we have presented and demonstrated the accurate MD of the CV and OAM modes based on a multi-view image for the polarization and mode degeneracy. By using the deep learning–based SPGD algorithm, the modal coefficients in simulation can be predicted with the average modal coefficient error of 0.416 %, and the time can be as short as 1.32 s, representing high calculation accuracy and efficiency. Due to the phase ambiguity of the SPGD algorithm, a set of intensity distributions may correspond to two sets of modal coefficients expressed in CV mode bases, where modal weights are the same but the phase is opposite to each other. Afterward, it can lead to different modal weights and phases in the OAM mode bases. Thus, the interference patterns are measured to determine the phase expressed in CV mode bases. In reality, distinguishing the direction of elliptical polarization is also beneficial to confirm the modal phase by using the QWP. Especially, for a single CP-OAM mode, the ambiguity of phase and circular polarization should be identified simultaneously. In the actual experiment, the generated beams have been accurately decomposed in the CV, LP-OAM, and CP-OAM mode bases with the highest purity of 99.5 % in CV modes, indicating high feasibility of the MD. By varying the optimized voltages of the EPC with high speed response and precise control, the CV modes with high purity have been experimentally generated and modulated every 4 s. Through invariant wave plates, switching the vortex modes has also been achieved, which offers a new method for manipulating vortex modes. As a result, a hybrid first-order PS may be constructed to describe the relationship among these three mode bases from the perspective of modal proportions. More importantly, it is expected to realize the intelligent generation of CV or OAM beams by measuring the exact proportions of the CV or OAM modes with programming and relevant intelligent techniques. It is believed that our works will provide a novel perspective on structure field analysis, easily modal control, and remote sensing.
